# *MYRF*-related mild encephalopathy with reversible myelin vacuolization: a case report and literature review

**DOI:** 10.3389/fgene.2023.1284060

**Published:** 2023-12-13

**Authors:** Shumei Yao, Xiufeng Mo, Changjiang Luo, Chuanqiang Qu

**Affiliations:** ^1^ Department of Neurology, Shandong Provincial Hospital, Shandong University, Jinan, China; ^2^ Department of Neurology, Jinan Shizhong District People’s Hospital, Jinan, China; ^3^ Department of Neurology, Shandong Provincial Hospital Affiliated to Shandong First Medical University, Jinan, China

**Keywords:** *MYRF*-related mild encephalopathy with reversible myelin vacuolization, infection, seizures, corpus callosum demyelination, genetic analysis

## Abstract

**Background:** *MYRF*-related mild encephalopathy with reversible myelin vacuolization (MMERV) is an inherited neurological disorder characterized by dysfunction in the central nervous system and widespread reversible leukoencephalopathy. This paper presents a confirmed case of familial MMERV and summarizes pertinent features to offer guidance for future diagnosis and treatment of MMERV.

**Case Introduction:** We have diagnosed a case of MMERV based on a history of seizures during early childhood and recurrent speech fluency issues in adulthood, reversible abnormal intensities in bilateral white matter in the centrum semiovale and corpus callosum, and the identification of myelin regulatory factor (*MYRF*) heterozygous variants.

**Conclusion:** *MYRF*-related mild encephalopathy with reversible myelin vacuolization is a rare autosomal dominant genetic disease, with early clinical manifestations often being seizures. The definitive diagnosis of MMERV can be confirmed through genetic analysis. Minimizing infections can help reduce disease recurrence. However, future research should explore the impact of *MYRF* heterozygous variants in the wider MMERV population.

## 1 Introduction

*MYRF*-related mild encephalopathy with reversible myelin vacuolization (MMERV) is a rare genetic disorder caused by missense mutations in myelin regulatory factor (*MYRF*), which acts as a transcriptional regulator of the myelin gene (Kurahashi et al., 2018). The disease was first described by Kurahashi et al. (Kurahashi et al., 2018). Currently, knowledge regarding MMERV has been based on two familial MMERV, which may be underdiagnosed due to limited awareness of the disease among clinicians and the fact that genetic testing technology is not yet fully available. Infectious diseases in children are a major triggering factor for MMERV (Imamura et al., 2010; Kurahashi et al., 2018; Saito et al., 2022). The clinical manifestations of MMERV include seizures, impaired speech (such as lack of fluency, dysarthria, and language difficulties), recurrent episodes of impaired consciousness (confusion or delirium), spontaneous motor difficulties, and limb numbness (Imamura et al., 2010; Kurahashi et al., 2018; Saito et al., 2022). Magnetic Resonance Imaging (MRI) of the brain showed abnormal intensities in bilateral white matter in the centrum semiovale and corpus callosum on diffusion-weighted, T2-weighted and apparent diffusion coefficient images.

Diagnosis of MMERV relies on recurrent nonspecific neurological symptoms and characteristic neuroimaging. Genetic analysis is necessary for confirming the diagnosis. Neuroimaging and genetic testing are crucial in differentiating MMERV from other conditions, such as cytotoxic lesions of the corpus callosum/mild encephalopathy with reversible splenic lesions (CLOCC/MERS) and X-linked Charcot-Marie-Tooth type 1 (CMTX1). Currently, the treatment options for MMERV are limited and based on hormonal steroids therapy with full recovery of symptoms within 1 week.

In this report, we describe a Chinese patient with familial MMERV. The patient presented only with a history of seizures during early childhood and speech fluency issues with/without seizures in adulthood. To enhance our understanding of this rare genetic disorder, we conducted a literature review, analyzing laboratory tests, imaging reports, and relevant literature to examine the pathogenesis, clinical presentation, imaging features, and treatment options associated with MMERV.

## 2 Case report

**FIGURE 1:**
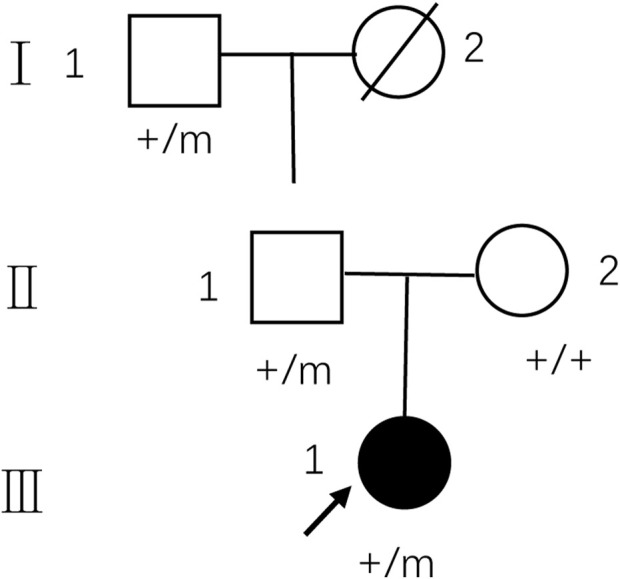
Pedigree of the family with MMERV. In the family trees, +/+ denotes normal individuals with two wild-type alleles, whereas +/m denotes heterozygous carriers of c.1208A>G in MYRF. Squares indicate males; circles indicate females; black indicate the proband of the family. An arrow indicates propositus. Slash marks indicate subjects who are deceased. Roman numerals indicate generations and Arabic numbers indicate subjects. MMERV, MYRF-related mild encephalopathy with reversible myelin vacuolization.

**FIGURE 2:**
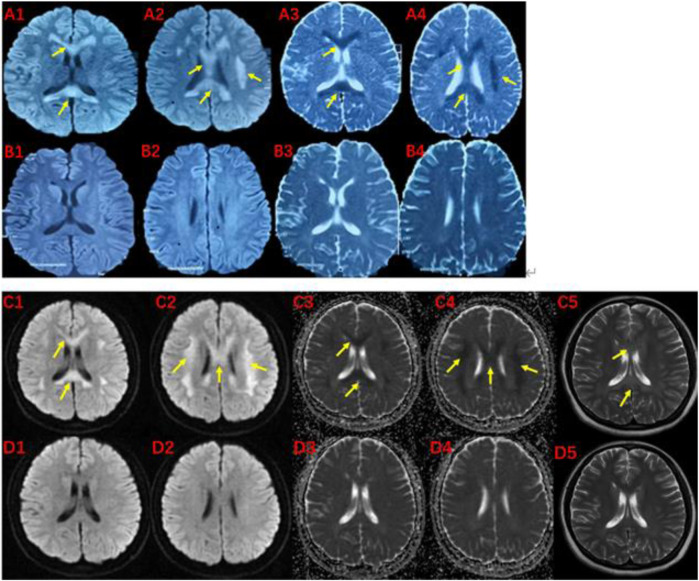
MRI of the proband (III-1) before and after the onset of the disease at 19 **(A, B)** and 27 **(C, D)** years of age.The brain MRI showed high intensities in bilateral white matter in the centrum semiovale and corpus callosum on diffusion-weighted (A1,A2,C1,C2), T2-weighted (C5), and low intensities on apparent diffusion coefficient images (A3,A4,C3,C4). These abnormal intensities disappear within two weeks (B1-B4 and D1-D5). The yellow arrow indicates involved structures.

The pedigree of the family is depicted in Figure 1. The proband (III-1) was a 27-year-old female with a history of two seizures after a fever in early childhood, but specific details were unknown. The grandfather and father of the proband also had a suspected history of seizures in childhood. The patient had a sudden onset of recurrent episodes of impaired speech fluency at the age of 19, which lasted for several hours and then resolved spontaneously. These episodes were exacerbated by no apparent trigger and were accompanied by gaze in both eyes (specific directions not available), right-sided mouth, closed teeth, stiff neck, and preserved consciousness during the episodes. Physical examination was negative for all pathological signs. Brain MRI showed abnormal intensities in bilateral white matter in the centrum semiovale and corpus callosum on diffusion-weighted, T2-weighted and apparent diffusion coefficient images, consistent with an acute demyelinating lesion (Figures 2A). Full-length sequencing of mitochondrial DNA was negative. The patient received steroids therapy with dexamethasone 10 mg and methylprednisolone 500 mg to alleviate symptoms, which did not recur. After 13 days, the cranial MRI showed complete normalization of the lesion (Figures 2B). After this infection with coronavirus at the age of 27, the patient developed fever up to 38.8°C, accompanied by generalized muscle aches and weakness. Three days after the infection, she experienced another episode of impaired speech fluency lasting more than 10 min, subsiding afterwards.

Neurological physical examination: clear and articulate speech, normal light reflexes in both pupils, normal muscle strength and tone in all limbs, normal tendon reflexes in upper limbs, no tendon reflexes elicited in lower limbs, no sensory abnormalities, normal ataxic movements, negative Romberg sign, normal gait, and no pathological signs elicited.

**FIGURE 3:**
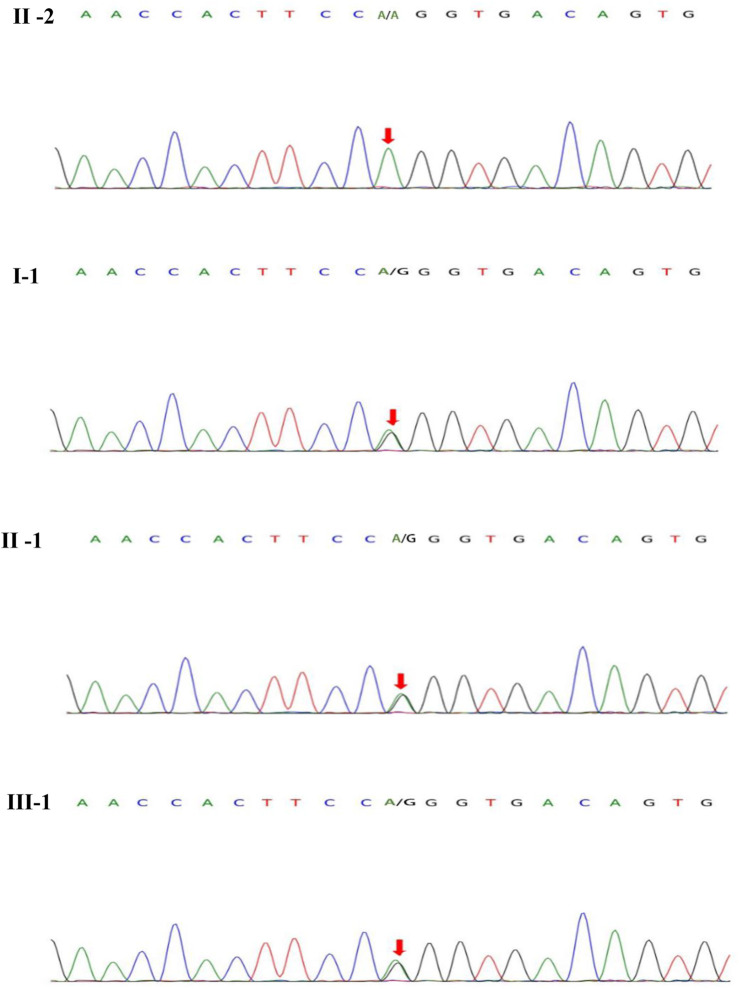
*MYRF* gene sequeneing results. II-2 are healthy individuals, the rest (I-1, II-1, III-1) are mutation carriers.

On blood, urine and stool tests, liver and renal function, thyroid function, blood biochemistry (including electrolytes such as potassium, sodium and chloride), full viral series, ANCA, antiphospholipid antibodies, homocysteine, C-reactive protein, erythrocyte sedimentation rate, glycated hemoglobin, and urine and stool routine were normal. Electrocardiography (ECG), echocardiogram, abdominal ultrasound, gynecological ultrasound and chest CT were also normal. The abnormal laboratory findings were as follows: white blood cell count:3.44*10^9/L, lymphocyte count: 0.83*10^9/L.The total T-cell count was 446.36/ul, T-helper cell count was 262.32/ul, T-suppressor/cytotoxic cell count was 136.35/ul and total B-cell count was 49.38/ul.The video electroencephalogram (EEG) showed a slight enhancement of slow waves in the theta band. Brain MRI showed abnormal intensities in bilateral white matter in the centrum semiovale and corpus callosum on diffusion-weighted, T2-weighted and apparent diffusion coefficient images, consistent with an acute demyelinating lesion (Figures 2C). We initially considered mild encephalopathy with reversible splenic lesions (MERS) due to the presence of neurological symptoms and extensive central nervous system demyelinating changes following the infection; However, considering that two immediate relatives had a history of seizures, we were unable to exclude the possibility of a genetic disorder. Therefore, along with general examination, we performed genetic testing. Whole exome sequencing of the proband revealed a missense mutation (*MYRF*- Q403R) at codon 403 of the *MYRF* gene: c.1208A>G (p.Gln403Arg) (Figure 3), resulting in an amino acid change from glutamine to arginine. The latest version of the Criteria and Guidelines for Interpretation of Genetic Variants (Richards et al., 2015) published by the American College of Medical Genetics and Genomics (ACMG) determined that the variant is likely pathogenic and is registered as NM_001127392: c.1208A>G(p.Gln403Arg). Meanwhile, the bioinformatics software REVEL predicted this variant as deleterious with a predicted value of 0.896.

Based on the patient’s history, clinical presentation, neurological examination, cranial MRI and genetic testing, the diagnosis of MMERV was made. Based on previous experience in the treatment of demyelinating diseases and the available evidence-based medical evidence, we treated with steroids regimen as follows: methylprednisolone 500 mg intravenously once a day for 3 days, there after the dose ladder was halved sequentially for 3 days each, and when it was reduced to less than 120 mg, it was switched to oral prednisone acetate 60 mg, still halving each dose for 3 days until discontinuation of the drug. Patient had no adverse reaction during treatment. The brain MRI lesion was completely normalized after 1 week (Figures 2D). Two carriers of the mutation were identified: Individual I-1 (grandfather of the proband) and Individual II-1 (father of the proband).

## 3 Discussion and conclusion

In the present study, we report the first confirmed familial case of MMERV in China based on clinical symptoms, reversible neuroimaging features and detection of missense mutations in the *MYRF* gene.

### 3.1 Genetic features

MMERV is a rare genetic neurological disorder caused by mutations in the *MYRF* gene, located on chromosome 11 (Kurahashi et al., 2018). *MYRF* is a membrane-bound transcription factor on the endoplasmic reticulum (ER), which encodes a homotrimeric membrane protein (Garnai et al., 2019; Wang et al., 2022). The N-terminal of this protein is autoproteolytically cleaved and translocated to the nucleus as a obligate homo-trimers, where it activates the transcription of the myelin gene (Emery et al., 2009; Garnai et al., 2019; Wang et al., 2022), which is crucial for oligodendrocyte differentiation and maintenance of myelin (Koenning et al., 2012). *MYRF* biochemistry relies on two fundamental mechanisms: auto-cleavage and the formation of obligate homo-trimers with the *MYRF* N-terminal fragments (Fan et al., 2021). Deleterious *MYRF* mutations specifically disrupt these mechanisms, leading to disease. In mature oligodendrocytes, conditional knockdown of *MYRF* results in a dramatic downregulation of myelin gene expression and myelin damage (Wang et al., 2022). Mutations in the structural domain of this gene may have an impact on myelin transcription.

In this study, we report a familial case with a history of neurological disorders, showing an autosomal dominant pattern of inheritance. A variant in the *MYRF* gene in this patient and two of her immediate relatives resulted in a change in amino acid from glutamine to arginine. Both glutamine and arginine are hydrophilic amino acids, but they differ in their acid-base properties. Glutamine is a neutral amino acid that exhibits stability in acidic and alkaline substances, while arginine is a basic amino acid and shows high solubility in acids. Substitutions of glutamine with arginine involve amino acids that possess distinct chemical properties, leading to disruptions in the structure and/or properties of the polypeptide chain, which subsequently manifests as a mutant phenotype. The variant impacts the DNA-binding domain of the *MYRF* gene, which is highly conserved. Luciferase analysis revealed a significant reduction in the transcriptional activity of the N-terminal region of *MYRF* due to this variant (Kurahashi et al., 2018), which subsequently affects the transcription of the myelin gene. These findings support a causal relationship between the c.1208A>G mutation in *MYRF* and encephalopathy with reversible myelin vacuolation, which may contribute genetically to the development of MMERV.

Kurahashi (Kurahashi et al., 2018) identified this missense mutation in the *MYRF* gene through genetic testing of nine symptomatic individuals from two unrelated families. In one family, the pedigree showed an autosomal dominant mode of inheritance, while the other family was too small to confirm. In all three familial cases of MMERV, carriers of this mutation showed normal psychomotor development under physiological, indicating relatively preserved *MYRF* function under normal conditions. This may be for the following reasons by Fan (Fan et al., 2021): the N-terminal fragment demonstrated enhanced stability in maintaining the homotrimeric structure required for transcription initiation. Additionally, the N-terminal homotrimer can tolerate more mutant fragments to activate the transcription of myelin genes, and its transcriptional activity is significantly higher than that other MRYF mutants. However, under pathological conditions such as infection, *MYRF* function is further impaired or fails to increase the transcriptional rate of myelin. This leads to myelin fragmentation and subsequent phagocytosis by activated microglia/macrophages in the white matter (Koenning et al., 2012), resulting in associated neurological symptoms and rapid, reversible severe demyelination manifestations on neuroimaging. Nevertheless, there are no irreversible neurological consequences following recurrent neurological events. Therefore, it can be concluded that the impact of this mutation on *MYRF* function is not severe enough to cause significant myelin-related diseases.

Recent studies in human disease suggest that deleterious *de novo* variants in *MYRF* are associated with congenital diaphragmatic hernia, cardiac anomalies including Scimitar syndrome, urogenital anomalies, and ocular development, consistent with expression in a range of tissues (Emery et al., 2009; Garnai et al., 2019). Haploinsufficiency (*MYRF*-G435R) and dominant negative functionality (*MYRF*-F387S,*MYRF*-Q403H or *MYRF*-L479V) of *MYRF* can lead to genetic syndromes in the above systems (Fan et al., 2021). The specific functional impairment depends on the effect of the variant of *MYRF* on the relevant proteins, either as a single symptom or as a multi-organ symptom.

### 3.2 Clinical features

The clinical manifestations of MMERV are diverse and nonspecific, lacking uniform diagnostic criteria. Predominantly, the symptoms in this family are characterized by seizures and speech fluency issues. Table 1 provides a summary of three familial MMERV symptom presentations.

**TABLE 1:** Familial MMERV symptom presentations (family A and B refer to the original report of Kurahashi et al., family C refers to that herein reported). Upper case Roman numerals represent the number of generations in the family line, lower case Roman numerals represent specific individuals in a given generation. “Number” represent “the total numbers of neurological episodes” in each carriers.

Family	Carriers	Number	Triggers	Neurological symptoms	Treatment
A	II-1	4	Infectious fever	Spontaneous motor difficulties, dysarthria and mild impaired consciousness	Unknown
	II-3	1	Infectious fever	Spontaneous motor difficulties	Unknown
	III-2	1	Infectious fever	Language barriers and spontaneous motor difficulties	Unknown
	III-3	1	Infectious fever	Seizures and impaired consciousness	Unknown
	III-5	1	Infectious fever	Delirious behavior, including meaningless talking and laughter	Unknown
	Ⅳ-1	6	5 infectious fever	3 seizures and loss of consciousness,2 episodes of arm numbness and loss of consciousness,1 loss of consciousness	Methylprednisolone/Dexamethasone
B	II-1	1	Infectious fever	Seizures and impaired consciousness	Methylprednisolone
	II-2	1	Infectious fever	mild impaired consciousness	Dexamethasone
	II-3	1	Infectious fever	Seizures and impaired consciousness	Unknown
C	I-1	1	Unknown	Seizures	Symptomatic treatment
	II-1	1	Unknown	Seizures	Symptomatic treatment
	III-1	4	Infectious fever	2 Seizures; 2 lack of speech fluency	Methylprednisolone/Dexamethasone

MMERV is commonly triggered by childhood infectious fevers, and this patient experienced a relapse due to a recent coronavirus infection. Previously, Nakazawa (Saito et al., 2022) reported a case of MMERV recurrence caused by a novel coronavirus infection, exhibiting no significant differences in symptoms when compared to previous infections with influenza A virus and *Mycoplasma* pneumonia. Upon summarizing the symptoms of three familial MMERV cases, we determined that seizures were the most commonly observed initial symptom of the disease. Other notable clinical symptoms included impaired speech (such as lack of fluency, dysarthria, and difficulty with speech), recurrent episodes of impaired consciousness (confusion or delirium), as well as spontaneous motor difficulties and limb numbness. These symptoms may not always be present when the patient has an attack. Meanwhile, these symptoms were transient and largely recovered gradually within a week of treatment without any observed neurological sequelae. It is noteworthy that only three patients (II-1 and IV-1 in family A and III-1 in Family C) in the study experienced multiple episodes. However, it is uncertain whether these episodes were triggered by predisposing factors or if other carriers had asymptomatic episodes. Further investigation in clinical studies is necessary to shed light on this intriguing observation.

### 3.3 Neuroimaging

Laboratory tests for MMERV are generally not helpful in making a diagnosis. However, the disease exhibits distinct radiological features. Initially, Brain MRI showed abnormal intensities in bilateral white matter in the centrum semiovale and corpus callosum on diffusion-weighted, T2-weighted and apparent diffusion coefficient images, consistent with an acute demyelinating lesion (Figure 2). These extensive lesions have generally returned to normal within 1 week. Therefore, the presence of extensive myelin vacuolation on MRI should prompt consideration of the *MYRF* variant.

In humans, the corpus callosum (CC) serves as the largest forebrain connection, playing a crucial role in integrating and transmitting information between the cerebral hemispheres. This interhemispheric communication is essential for various cortical functions, including sensory integration, motor coordination, visuomotor processing, abstract reasoning, language comprehension, and emotional regulation (Pânzaru et al., 2022). Demyelination of the corpus callosum can lead to bilateral hemispheric disconnection, resulting in disruption of higher cortical functions. Previous reports have described patients with the *MYRF* variant who exhibited extensive white matter lesions involving the entire corpus callosum, surpassing the extent of lesions observed in our case. Correspondingly, these patients displayed more severe symptoms, including profound impairment of consciousness, compared to the current case.

### 3.4 Differential diagnosis

For an accurate diagnosis, genetic sequencing is required to confirm the presence of the *MYRF* mutation. However, most diseases are considered in view of the clinical presentation and imaging features. Therefore, we approach the differential diagnosis from this perspective.

CLOCC/MERS is the primary differential diagnosis for MMERV. CLOCC/MERS typically affects children or adolescents and is often secondary to infectious diseases like respiratory and gastrointestinal infections, but can also be secondary to non-infectious diseases such as Kawasaki disease. The main clinical features include central neurological disturbances like confusion, consciousness disruptions, and seizures, often accompanied by hyponatremia. These symptoms usually recover completely within a month (Kubo et al., 2022; Xue et al., 2022). Diagnosis heavily relies on brain MRI, where DWI reveals a transient high signal confined to the corpus callosum with reduced ADC, known as MERS type I. Other areas of the brain white matter may also show imaging abnormalities, known as MERS type II (Takanashi et al., 2010; Xue et al., 2022). There are no significant differences in clinical symptoms and prognosis between type I and type II patients (Xue et al., 2022). In contrast, MMERV presents clinically indistinguishable from MERS type II. Therefore, when MRI shows extensive myelin vacuolization, genetic analysis should be performed to determine the presence of *MYRF* variants to further diagnose the disease and guide eugenics in this family.

Additionally, CMTX1 can sometimes present as familial/recurrent transient leukoencephalopathy with reversible callosal and deep brain white matter lesions similar to MMERV. CMTX1 is caused by mutations in the GJB1 gene located on the X chromosome, which encodes connexin 32 (Cx32), a myelin-associated protein involved in the formation of gap junctions. Cx32 plays a vital role in the homeostasis of myelinated axons and is expressed in both the peripheral and central nervous systems (Tsai et al., 2016; Grosz et al., 2021). Many CMTX1 patients have been reported to experience transient leukoencephalopathy triggered by febrile infections, but their main clinical manifestations are motor and sensory symptoms (Mizuguchi et al., 2023), rather than seizures and disturbances of consciousness. Definitive diagnosis still depends on genetic testing.

### 3.5 Case management

Despite the lack of evidence for the efficacy of steroids therapy in MMERV, it is still widely used. Of the three MMERV families, only four proband explicitly received steroids therapy, the treatment regimens for the remainder of family A and B were unknown, and the two carriers in family C did not receive steroids therapy. However, it is worth noting that all patients made a full clinical recovery without any neurological sequelae. Furthermore, the last episode of the proband in family C were characterized by a recurrent lack of speech fluency, which was recovered before steroids therapy. This finding prompt us to consider the necessity of steroids therapy.

In conclusion, *MYRF*-related mild encephalopathy with reversible myelin vacuolization (MMERV) is a rare autosomal dominant neurological disorder characterized by early clinical manifestations mostly in the form of seizures. Neurologists should consider the diagnosis of MMERV when an infection is followed by symptoms of central neurological function and extensive reversible cerebral white matter lesions. The definitive diagnosis of MMERV can be established through genetic analysis. Minimizing the risk of infection can help reduce the recurrence of the disease. However, future research should explore the impact of *MYRF* heterozygous variants in the wider MMERV population.

## Data Availability

The data presented in the study are deposited in the Sequence Read Archive (SRA) repository, accession number PRJNA1043752.
